# The Differential Diagnosis between Pseudo Cushing's Syndrome and True Cushing's Syndrome in a Septic Patient in the Pre-Agonal Phase: A Case Report

**DOI:** 10.2174/0118715303346706250206070813

**Published:** 2025-03-04

**Authors:** Simone Antonio De Sanctis, Sabrina Chiloiro, Eloisa Sofia Tanzarella, Filippo Bongiovanni, Antonella Giampietro, Amato Infante, Gennaro De Pascale, Laura De Marinis, Massimo Antonelli, Alfredo Pontecorvi, Antonio Bianchi

**Affiliations:** 1 Department of Endocrinology, UOC Endocrinologia e Diabetologia, Fondazione Policlinico Universitario A. Gemelli IRCCS, 00168, Roma, Italy;; 2 Dipartimento di Medicina Traslazionale, Università Cattolica del Sacro Cuore, Roma, Italy;; 3 Dipartimento di scienze biotecnologiche di base, cliniche intensivologiche e perioperatorie, Università Cattolica del Sacro Cuore, Roma, Italy;; 4 Dipartimento di scienze delle emergenze, anestesiologiche e della rianimazione, Università Cattolica del Sacro Cuore, Roma, Italy;; 5 Department of Radiology, UOC Radiologia d'Urgenza, Fondazione Policlinico Universitario Agostino Gemelli IRCCS, Università Cattolica del S. Cuore, Largo A. Gemelli, 8, 00168, Rome, Italy

**Keywords:** Pseudo-Cushing, sepsis, pre-agonal, adrenal, Cushing, differential diagnosis

## Abstract

**Introduction:**

Sepsis is an illness characterized by a high-stress condition for patients, accompanied by alterations in biochemical processes, behavior, and levels of consciousness. Hormonal alterations that can be seen in this context include increased plasma cortisol values, a condition known as pseudo-Cushing's syndrome (PCS), which in exceptional cases requires a differential diagnosis from true Cushing's syndrome (CS).

**Case Presentation:**

We report a septic patient with pseudo-Cushing's syndrome in the pre-agonal phase, suggesting that PCS during sepsis is an underestimated condition, as the severity of the patient's clinical condition is compounded by the difficulty of diagnosis itself.

**Conclusion:**

In this clinical case, for the severe clinical conditions of the patient and the poor prognosis, we conducted a comprehensive endocrine work-up to rule out an ACTH-dependent hypercortisolism that, if confirmed, could have changed the therapeutic approach and the prognosis of the reported patient.

## 1 INTRODUCTION

The pseudo-Cushing's syndrome (PSC) encompasses several disorders that can occur in high-stress situations and have clinical and biochemical features similar to those of Cushing's syndrome.

In true Cushing's syndrome, the hypersecretion of cortisol from the adrenal cortex may be adrenocorticotropic hormone (ACTH)-dependent or ACTH-independent.

Population studies examining the epidemiology of CS and PCS are limited; however, the annual incidence of CS ranges between 1.8 and 3.2 cases per million population [1], and it was found that Cushing's syndrome occurs mainly in females, with a female-to-male ratio of 4-5:1 except for prepubertal patients, where a strong male predominance has been observed and during the fourth decade of life [2]. In the former case, we may have an underlying ACTH hypersecretion by the pituitary gland (a condition known as 'Cushing's disease', which is the most common case with a prevalence of 1-10/100000 population) due to a pituitary neuroendocrine tumor (Pit-NET), or by a non-pituitary neuroendocrine tumor (NET), such as small-cell carcinoma of the lung or a carcinoid (a condition known as 'ectopic ACTH secretion syndrome').

ACTH-independent hypercortisolism usually results from exogenous corticosteroid administration or other oncological diseases, such as adrenal adenomas and adrenal carcinomas secreting cortisol. The annual incidence of benign adrenal adenomas and adrenal cancers is 0.6/million and 0.2/million, respectively [3].

PCS, on the other hand, is not caused by an organic disorder of the hypothalamic-pituitary-adrenal axis but by a heterogeneous group of para-physiological and pathological conditions, such as stress, major surgery, sepsis, agonal and pre-agonal phase of life or even very intense emotional stress, chronic alcoholism, withdrawal syndrome, major depression and poorly controlled diabetes mellitus, that lead to an increase in cortisol production acting secondarily on the aforementioned axis [4].

PCS is a relatively more frequent condition than CS (although its incidence is difficult to estimate), being underpinned by a variety of common clinical conditions, which can lead to hyperactivation of the corticotropic axis and the manifestation of phenotypic features similar to those of CS [5].

Discrimination between CS and PCS is often difficult because many symptoms and signs overlap in these conditions, although they are often more blurred in the latter case. These include central obesity with moon facies and buffalo hump, impaired glucose tolerance, skin bruising and striae rubrae, hypertension, acne, hirsutism, amenorrhoea, sexual dysfunction, recurrent infections, and osteoporosis. Moreover, the substantial overlap in the biochemical tests commonly used to diagnose both conditions makes it difficult for the physician to differentiate true CS, particularly if it is mild, from PCS [6].

In this study, we present a case of difficult differential diagnosis between CS and PCS in a septic patient.

## 2 CASE DESCRIPTION

A 74-year-old man was admitted to the emergency department of our hospital in August, 2023, for asthenia and worsening of dyspnoea that had occurred for a month, being associated with progressive hypotonia, muscular hypotrophy, cognitive deterioration, and weight loss (around 15 kg in the last six months). The patient had undergone previous diagnostic tests for organic deterioration, such as a total body contrasted computed tomography (CT) scan, that disclosed the presence of localized or systemic neoplasia. Previous medical history also reported impaired glucose tolerance in treatment with oral hypoglycemic drugs (metformin), major depression in treatment with mirtazapine and vortioxetine, and intraductal papillary mucinous neoplasia of the pancreas (IPMN) in follow-up.

At the clinical observation, clinical conditions were very poor, with severe dyspnoea, cachexia, sarcopenia, and marked hypotrophy of muscle masses. Body temperature was normal; blood pressure was 125/70 mmHg. An arterial haemogas analysis was performed in ambient air and demonstrated a severe respiratory acidosis, with severe hypoxia (pH 7.25, pO2 52 mmHg, pCO2 101 mmHg, Hb 18 g/dL, glycemia 232 mg/dL, lactate 2.8 mmol/L, HCO3- 35 mmol/L, sodium 145 mEq/L, potassium 3.9 mEq/L, calcium corrected for albumin 8.9 mg/dL, magnesium 2.6 mg/dl, and phosphorus 5.9 mg/dL). The patient was, therefore, transferred to the intensive care unit and treated with non-invasive mechanical ventilation for acute hypoxic-hypercapnic respiratory failure.

An urgent, not contrasted chest CT proved bilateral mid-basal pneumonia. Antibiotic therapy was empirically started and then modified to oxacillin and maxipime according to the results of antibiograms performed on blood cultures, showing a systemic infection due to Methicillin-sensitive *Staphylococcus Aureus* (MMSA) and *Klebsiella aerogenes*. Blood tests with blood count, reactive C-protein (198 mg/L), and procalcitonin (6.24 ng/mL) confirmed the diagnosis of sepsis.

A neurological consultant confirmed the severe hypotonia and the deficit of strength in the flexor muscles of the neck and four limbs. The reflexes were not evoked. No abnormalities of ocular motility, eyelid ptosis, and muscle or lingual fasciculations were detected. Electromyography of the deltoid, biceps brachii, and right rectus femoris muscles documented a myopathic recruitment pattern suggestive of sporadic late-onset nemaline myopathy (SLONM), a rarely acquired myopathy of unknown aetiology, typical of adulthood and characterized by rapid-onset proximal muscle weakness and the presence of nemaline bodies on muscle biopsy [7].

In order to rule out concomitant hypothyroidism and hypoadrenalism [8], basal hormone dosages were given, showing severe hypothyroidism (TSH 0.4 microIU/mL, fT3 1.1 pg/mL, fT4 3.0 pg/mL) and an ACTH-dependent hypercortisolism (basal ACTH: 88 pg/mL and cortisol 437 ng/mL).

We also performed a 24-hour urine collection to detect cortisol, which was found to be moderately elevated (150 micrograms/24 hours) but not to the degree that met the diagnostic criteria for CS syndrome [2].

**Fig. (1):**
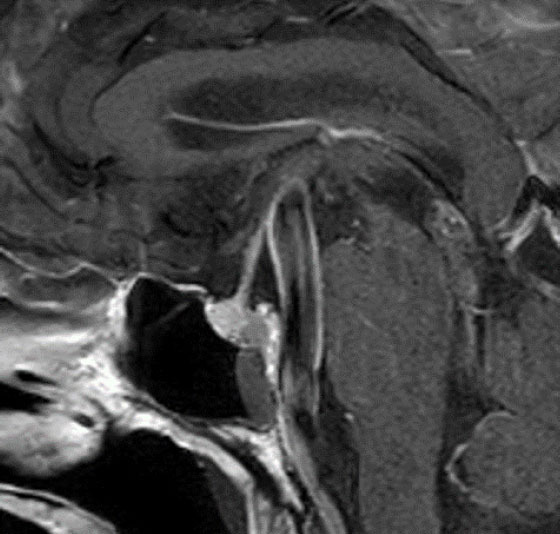
Sagittal high-resolution 1.5T T1w image after contrast medium shows the regular morphology of the pituitary gland, with the physiological distinction between adenohypophysis and neurohypophysis.

**Fig. (2):**
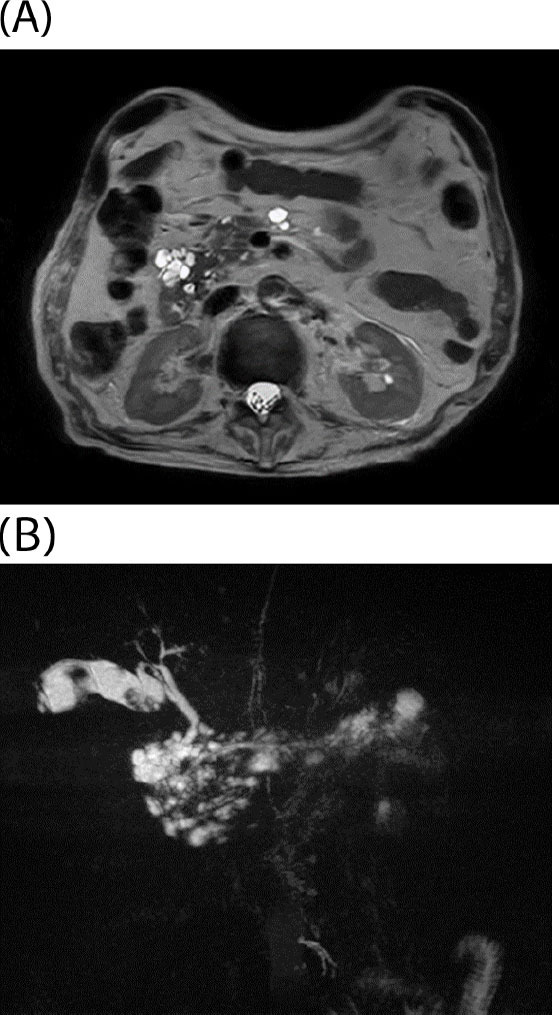
**(A)** Axial T2 image without contrast shows the region of the pancreatic head where the larger pancreatic cystic ectasias are evident; **(B)** MRCP image without contrast medium demonstrates the presence of multiple cystic formations spread throughout the pancreatic parenchyma and mild ectasia of the Wirsung duct, in relation to the IPMN condition.

Probably, the hypothyroidism, previously unrecognized in the patient’s medical history, was a result of his overall compromised health, commonly referred to as euthyroid-sick syndrome. Hormone replacement therapy with triiodothyronine and levothyroxine was, therefore, started. Moreover, pituitary magnetic resonance imaging with contrast medium showed a normal gland (Fig. **1**), ruling out any pituitary or brain mass lesions or abnormalities. An abdominal contrasted computed tomography (CT) and a magnetic resonance cholangiopancreatography (MRCP) scan confirmed stability for the dimension of the IPMN and excluded other macroscopically evident neoplasia (Figs. **2A** and **2B**).

According to the severe patient’s clinical condition, dynamic tests were performed to better define the aetiology of the hypercortisolism [2] and to rule out ectopic ACTH-secreting neuroendocrine tumors.

A low-dose dexamethasone suppression test (dexamethasone 1 mg overnight: Nugent's test) and a high-dose dexamethasone suppression test (dexamethasone 8 mg overnight: Liddle II test) showed the absence of cortisol inhibition, as reported in Table **1**, while ACTH was 91 pg/ml in the first exam and 94 pg/ml in the second. Therefore, the dexamethasone-suppressed corticotropin-releasing hormone (CRH) stimulation test ultimately suggested a diagnosis of non-neoplastic ACTH-dependent hypercortisolism [9], that was more suggestive of a PCS [10], as reported in Table **2**.

In this case, it was not necessary to perform other dynamic endocrine tests in order to exclude the possibility of true CS. During the hospitalization in the ICU, the patient’s respiratory function further worsened, an endotracheal intubation was performed, and inotropic drug therapy was introduced for the development of septic shock. Unfortunately, the patient developed a septic shock and multi-organ failure (MOF), and finally, he died. The autopsy examination confirmed the absence of pituitary and other neuroendocrine tumors that could explain the hypercortisolism and instead showed bilateral adrenal hypotrophy.

## 3 DISCUSSION

The discrimination between CS and PCS is often difficult because many symptoms and signs overlap. This clinical case is largely illustrative of the difficulty of correctly diagnosing the origin of hypercortisolism in a patient with a severe illness. Due to the difficult clinical conditions of the patient and the poor prognosis, we conducted a comprehensive endocrine work-up [11].

The finding of clinical and biochemical features suggestive of PCS syndrome does not always dictate the performance of the diagnostic algorithm. In this exceptional case, considering the severity of the patient's condition, we performed all diagnostic tests with a view to ruling out the diagnosis of CS syndrome, which would possibly have required medical or surgical treatment.

The differential diagnosis between PCS and CS in critical illnesses like sepsis and septic shock may have clinical significance in patients with a diagnosis in doubt, for whom a certain diagnosis is expected to change the therapeutic approach and the prognosis [12].

The tests performed during the patient's stay in the ICU were preparatory to the patient's diagnostic framing. Sepsis activates the hypothalamic-pituitary axis, increasing cortisol production [13]. However, it is also important to note that a true Cushing's syndrome can predispose individuals to septic status, in addition to the fact that it could have explained the patient's entire clinical presentation, particularly if it was associated with a pituitary adenoma or an ACTH-secreting ectopic tumor [14]. The above cases were ruled out by performing biochemical and hormone tests and radiological examinations, so the only possible explanation was that the patient had developed sepsis-induced PCS.

Sepsis is a life-threatening organ dysfunction caused by an unregulated response of a host. Septic shock is its most severe form. It is manifested by a drop in blood pressure, which decreases tissue perfusion pressure, causing hypoxia that is characteristic of shock and shock is one of the conditions that prelude agony [15]. We can define agony as the final phase of life: it coincides with the period immediately preceding death, which can last from a few hours to even one or two days. More specifically, it is possible to make a distinction between the pre-agonal phase and agony proper; the pre-agonal phase precedes the agonist phase and is characterized by worsening general conditions, refusal to eat and particular behavioural signs (including refusal to speak, psycho-motor agitation and assumption of the fetal position); the agonist phase is characterized by disturbances of consciousness (*e.g.* increased drowsiness), reduction or disappearance of muscle tone, respiratory disorders of various kinds, and circulatory disorders [16].

The agonic and pre-agonic phases are, therefore, dense with stressful events, which can cause changes at a pathophysiological and hormonal level in the patient. These include activation of the hypothalamic-pituitary-adrenal (HPA) axis with consequent hypercortisolism, which identifies the well-known picture of pseudo-Cushing's syndrome. Several studies have now reported that the rise in cortisol in response to critical illness in a patient in the agonic or pre-agonic phase is driven primarily by peripheral adaptations of the body rather than by central hypothalamic-pituitary alterations [17]. These include a reduction in cortisol-binding proteins (with a consequent increase in the free portion of the hormone), a reduction in the hepatic and renal catabolism of cortisol itself, a prolongation of its half-life, and changes in receptor expression. These are, therefore, the mechanisms underlying the increase in plasma cortisol concentration, even in patients in the pre-agonist phase during septic shock, as in the case we have described [18]. As already mentioned, sepsis is one of the possible causes that increase cortisol production. However, the data available in the literature suggest that in patients with septic shock, the determination of a single plasma cortisol value has no predictive value. It is also true, however, that the degree of this increase can be extremely variable. This variability may, in part, be related to the type of infection, the duration of the shock, and the patient's blood pressure at the time the blood sample was taken. Interestingly, most critically ill patients admitted to the ICU have substantially elevated plasma cortisol concentrations that are proportional to the severity of the illness [19].

However, there is no indication that plasma cortisol concentration, especially when measured in a single blood draw, can be used in predicting patient outcomes [20].

Previous evidence underlined that during the acute phase of stress, such as that associated with systemic infection, the HPA axis is activated mainly by CRH-independent pathways involving immune mediators [21]. The influence of many cytokines on the secretory activity of the HPA axis has been demonstrated, the main ones being IL-1, IL-6, and TNF-α, which increase the secretion of CRH and ACTH [22, 23].

Experiments conducted both *in vitro* and *in vivo* in various animal and human models have shown that sepsis itself can alter both directly and indirectly the HPA axis response. A typical neuroendocrine response to systemic infection-related stress includes increased secretion of catecholamines by the sympathetic nervous system at the level of the adrenal medulla, release of CRH and vasopressin by the hypothalamus into the portal circulation, and secretion of oxytocin from the posterior part of the pituitary gland, with subsequent secretion of ACTH by the anterior pituitary cells, followed within seconds by increased plasma glucocorticoid levels. Direct damage to neuroendocrine cells, which disrupts this axis, along with the activation of mechanisms that increase peripheral tissue resistance to cortisol, results in an inadequate response. Therefore, patients in septic shock often require glucocorticoid replacement therapy [24]. The pathophysiology of the HPA axis during sepsis is, therefore, an extremely complex subject and further studies are needed to fully understand its dynamics [25].

## CONCLUSION

In conclusion, our clinical case describes a septic patient with pseudo-Cushing's syndrome in the pre-agonal phase, suggesting that PCS during sepsis is an underestimated condition, as the severity of the patient's clinical condition is compounded by the difficulty of diagnosis itself.

The clinical case we described shows that PCS syndrome, being related to intense stress, maybe a common finding in the septic patient in the pre-agonal phase. This needs to be kept in mind since, in clinical practice, endocrinologic consultation is often requested in intensive care or internal medicine departments for this type of patient, and it is important to distinguish, therefore, the true CS syndrome from the PCS syndrome. The differential diagnosis between PCS and CS in critical illnesses like sepsis and septic shock may have a clinical significance in patients with a diagnosis in doubt, for whom a certain diagnosis is expected to change the therapeutic approach and the prognosis, for example, with a view to starting supportive therapy in an already critical patient. In this context, it is necessary to make a diagnosis in a timely manner, especially to rule out CS syndrome. In fact, in case it was a CS syndrome, there would probably have been an indication to undertake medical therapy for hypercortisolism.

## Figures and Tables

**Table 1:** ACTH and cortisol levels in basal conditions and dynamic tests.

Basal ACTH levels	88 pg/mL
Basal cortisol levels	437 ng/mL
Cortisol after 1 mg dexamethasone overnight	649 ng/ml
Cortisol after 8 mg dexamethasone overnight	357 ng/mL
24 hours Urinary Free Cortisol	144 mcgr/24 hours

**Table 2:** Plasmatic cortisol levels during dexamethasone suppressed corticotropin-releasing hormone stimulation test. A plasmatic cortisol level > 14 ng/mL identify ACTH-dependent hypercortisolism; a plasmatic cortisol level <14 ng/mL identify pseudo-cushing conditions.

-	Basal	After 15 minutes	After 30 minutes	After 45 minutes	After 60 minutes
Cortisol levels ng/mL	7	9	10	8	7

## Data Availability

The data and supportive information are available within the article.

## LIST OF ABBREVIATIONS

PCS: Pseudo-Cushing's Syndrome
CS: Cushing's Syndrome
Pit-NET: Pituitary Neuroendocrine Tumor
NET: Non-pituitary Neuroendocrine Tumor
SLONM: Sporadic Late-onset Nemaline Myopathy
CT: Computed Tomography
MRCP: Magnetic Resonance Cholangiopancreatography
CRH: Corticotropin-Releasing Hormone
HPA: Hypothalamic-Pituitary-Adrenal

## STANDARDS OF REPORTING

CARE guidelines were followed.
